# Why does oriental arborvitae grow better when mixed with black locust: Insight on nutrient cycling?

**DOI:** 10.1002/ece3.3578

**Published:** 2017-12-06

**Authors:** Xuedong Chen, Ming Tang, Xinlu Zhang, Chantal Hamel, Wei Li, Min Sheng

**Affiliations:** ^1^ State Key Laboratory of Soil Erosion and Dryland Farming on the Loess Plateau Northwest A&F University Yangling Shaanxi China; ^2^ College of Life Science Northwest A&F University Yangling Shaanxi China; ^3^ College of Forestry Northwest A&F University Yangling Shaanxi China; ^4^ Quebec Research and Development Centre Agriculture and Agri‐Food Canada Quebec QC Canada

**Keywords:** arbuscular mycorrhizal fungi, mixture, *Platycladus orientalis*, *Robinia pseudoacacia*, soil properties

## Abstract

To identify why tree growth differs by afforestation type is a matter of prime concern in forestry. A study was conducted to determine why oriental arborvitae (*Platycladus orientalis*) grows better in the presence of black locust (*Robinia pseudoacacia*) than in monoculture. Different types of stands (i.e., monocultures and mixture of black locust and oriental arborvitae, and native grassland as a control) were selected in the Loess Plateau, China. The height and diameter at breast height of each tree species were measured, and soil, shoot, and root samples were sampled. The arbuscular mycorrhizal (AM) attributes, shoot and root nutrient status, height and diameter of black locust were not influenced by the presence of oriental arborvitae. For oriental arborvitae, however, growing in mixture increased height and diameter and reduced shoot Mn, Ca, and Mg contents, AM fungal spore density, and colonization rate. Major changes in soil properties also occurred, primarily in soil water, NO_3_‐N, and available K levels and in soil enzyme activity. The increase in soil water, N, and K availability in the presence of black locust stimulated oriental arborvitae growth, and black locust in the mixed stand seems to suppress the development of AM symbiosis in oriental arborvitae roots, especially the production of AM fungal spores and vesicles, through improving soil water and N levels, thus freeing up carbon to fuel plant growth. Overall, the presence of black locust favored oriental arborvitae growth directly by improving soil water and fertility and indirectly by repressing AM symbiosis in oriental arborvitae roots.

## 1 INTRODUCTION

Forests play an important role in producing timber, fuel, and pulp wood and supporting ecosystem functions and services such as soil erosion control (Lang et al., 2014). Afforestation, often established as monocultures and mixtures, is an effective way to improve degraded areas (Lang et al., 2014; Zhang, 2005). Some evidence indicates that mixtures have been more effective than monocultures at improving soil fertility and nutrient cycling (Binkley, Dunkin, DeBell, & Ryan, 1992; Montagnini, 2000), stand productivity (DeBell, Cole & Whitesell, 1997; Bauhus, van Winden, & Nicotra, 2004; Forrester, Bauhus, & Cowie, 2005; Groninger, Zedaker, & Fredericksen, 1997), carbon sequestration (Kaye, Resh, Kaye, & Chimner, 2000; Parrotta, 1999; Resh, Binkley, & Parrotta, 2002), and tree nutrition status and resistance to pests and diseases (Lamb, 2011). However, efficacy has varied by tree species (Carnevale & Montagnini, 2002; Groninger et al., 1997; Guariguata, Rheingans, & Montagnini, 1995; Kelty, 2006; Montagnini, Ugalde, & Navarro, 2003), and if one species suppresses the growth of the other, a mixed stand may be less productive than a monoculture (Forrester et al., 2005). Tree productivity is a function of the supply, capture, and efficiency of use of resources, and species interactions in mixed‐species stands can affect each of these factors (Richards, Forrester, Bauhus, & Scherer‐Lorenzen, 2010). Thus, identifying the factors being responsible for changes to tree productivity in mixed‐species tree plantations is a matter of prime concern in forestry.

Arbuscular mycorrhizal (AM) fungi are a key functional group of soil microbes (Álvarez‐Sánchez, Sánchez‐Gallen, Hernández‐Cuevas, Hernández, & Cruz, 2016; Wahbi et al., 2016). Early colonization of plant roots by AM fungi is crucial for the success of an afforestation plan, because AM symbiosis plays an important role in improving plant establishment and growth (Alguacil, Torres, Torrecillas, & Roldán, 2011; Smith & Read, 2008). Conversely, the host plants and soil conditions, such as water content and fertility, can influence the growth and functions of AM fungi (Bencherif, Boutekrabt, Dalpé, & Sahraoui, 2016; Chaiyasen, Chaiya, Douds, & Lumyong, 2016; Legay et al., 2016; Lekberg & Waller, 2016). Afforestation changes the vegetation cover, causing an impact on soil microclimatic conditions and physicochemical properties (Antoninka, Reich, & Johnson, 2011; Su & Guo, 2007; Yang et al., 2013; Zheng et al., 2014), which means there is considerable potential for afforestation to affect the growth of resident AM fungi and their beneficial effects on host plants. A better understanding of the effects of afforestation type on AM symbiosis development is essential to improve timber yield and provision of ecosystem services.

The Loess Plateau in western China is a region with distinctive topographical and geological features (Peng & Yu, 1995). This area is famous for its deep loess. Due to the special geographic landscape and long history (over 5,000 years) of anthropogenic disturbance, the vegetation cover was diminishing, exposing the soil to erosion (Zhang, 2000). Restoring the vegetation should therefore improve the ecological conditions (Zhang, 2005). Black locust (*Robinia pseudoacacia*), an N‐fixing legume tree, has a strong capacity to increase soil N levels (Rice, Westerman, & Federici, 2004; Tateno et al., 2007). Oriental arborvitae (*Platycladus orientalis*) is a conifer with an expansive root system and a long lifespan (Li & Liu, 2013). Over the last three decades, both black locust and oriental arborvitae have been widely planted as monocultures to control soil erosion. Several researchers had reported shifts in soil properties under black locust and oriental arborvitae monocultures (Liu, Duan, Liu, & Feng, 2009; Song, Lou, & Hou, 1995; Xue, Liang, & Yang, 2000), and therefore, more and more mixtures of black locust and oriental arborvitae have been established in recent years. Groninger et al. (1997) found that intercropping conifers and black locust provide greater economic flexibility, biological diversity, and aesthetic benefits. In the Loess Plateau, oriental arborvitae also grows better in the presence of black locust than in monoculture, whereas the reasons for which are still unclear. Normally, all the soil properties, AM symbiosis development, and plant nutrition play an important role in tree productivity (Forrester et al., 2005; Gorzelak, Asay, Pickles, & Simard, 2015; Rothe & Binkley, 2001; Tang & Ohsawa, 2002), and thus, we suggest that the benefit of mixed planting maybe stem from afforestation‐type‐induced changes in soil properties, AM symbiosis development, and plant nutrition. In order to understand how the growth of oriental arborvitae is modified by afforestation type, it is therefore necessary to simultaneously consider tree growth, soil properties, AM symbiosis development, and plant nutrient status, as well as the interactions among these variables.

Our study was conducted at a long‐term experimental site established in 2003 in the Loess Plateau, in western China, to evaluate the effects of different afforestation types (black locust monoculture, oriental arborvitae monoculture, black locust × oriental arborvitae mixture, and native grassland as a control) on soil erosion. Here we wanted to explicitly test how soil properties, AM symbiosis development, plant nutrient status, and growth varied with afforestation types. We hypothesized that black locust in mixture improved oriental arborvitae growth through changing soil properties, AM symbiosis development, and plant nutrition. We addressed two questions: Do soil properties, AM symbiosis development, and plant nutrition vary with afforestation types and are these changes related to oriental arborvitae growth?

## 2 MATERIALS AND METHODS

### 2.1 Experimental site

The study was conducted at the Changwu Soil and Water Conservation Research Station of the Chinese Academy of Sciences in the Wangdonggou watershed, Shaanxi Province, in northwest China (longitude 107°42′ E, latitude 35°12′ N; elevation 1,206 m). The study area, located in the southern part of the Loess Plateau, is characterized by a warm temperate subhumid continental climate, with an annual average temperature of 9.4°C, mean annual precipitation of 575 mm, and a frost‐free period of 171 days (China Meteorological Data Service Center). The groundwater level is about 50–80 m below the soil surface, which precludes upward capillary flow into the root zone. The soil at the study site has a silty clay loam texture and was mostly covered by native grasses (such as *Bothriochloa ischaemum*,* Arundinella hirta*,* Artemisia argyi*). Pure stands of black locust and oriental arborvitae and their 50:50 mixtures were established at a 2 × 2 m spacing on the native grassland in 2003, to control soil erosion. To prevent the slope aspect and differences in the initial soil properties from influencing tree growth, all planted areas were selected for uniformity in slope (35°) and soil properties and therefore had the same aspect. The native grassland used as a control was left undisturbed, and the areas were fenced off after the trees were planted, to prevent further anthropogenic disturbance.

### 2.2 Shoot, root, and soil sampling

In 2013, that is, 10 years after planting, five stands were selected for each of afforestation types (black locust monoculture, oriental arborvitae monoculture, black locust × oriental arborvitae mixture, and native grassland as a control), and a 20 m × 20 m sampling plot was set up in each of these stands. The distances between plots were ranged from 150 m to 700 m, and this area between plots was used as a buffer to prevent root invasion.

In each native grassland sampling plot, five soil cores (8 cm in diameter) were taken in the 0‐ to 20‐cm soil layer using a handheld power sampler and pooled into a single composite sample. In each of tree planted plot, five trees of each species were randomly selected; tree height and diameter at breast height were recorded as nondestructive measurements; shoot samples were cut using handheld scissors; and soil cores (8 cm in diameter) close to the tree trunk (at distance <50 cm) were collected in the 0‐ to 20‐cm soil layer using a handheld power sampler. All of these samples were transported on ice to the laboratory, where the soil cores and shoot samples from a given tree in each plot were pooled prior to analyses. Roots in the soil cores were shaken to detach loose soil and were then carefully brushed to collect rhizosphere soil. The roots were rinsed in running water, collected on a 0.2‐mm mesh sieve, separated from debris by hand, and dried with paper towels. The root and soil samples were stored in a freezer at −20°C prior to processing.

### 2.3 Soil properties

Soil water content was determined by drying 10‐g samples at 105°C until a constant mass was reached (AOAC 1990). Soil organic matter was measured using the potassium dichromate volumetric method (Nelson & Sommers, 1982). Available P was extracted in a 0.5 mol/L NaHCO_3_ solution and quantified using the molybdenum–antimony colorimetric method (Page, 1982). Nitrate–nitrogen (NO_3_‐N) was quantified on an AA3 Continuous Flow Analytical System (Bran+Luebbe AA3, Germany) after extraction in 1 M KCl (Hu et al., 2016). Available K, Cu, Zn, Fe, Mn, Ca, and Mg were quantified by flame atomic absorption spectrometry (FAAS; AA‐7003A, Beijing, China) using the methods of the AOAC (1990) and Jorhem (2000). Soil catalase activity was determined using the KMnO_4_ titrimetric method (Roberge, 1978). Alkaline phosphatase and dehydrogenase activity were measured using the methods described by Tabatabai (1982).

### 2.4 AM fungal spore density

The AM fungal spores were extracted from 15‐g frozen soil samples by means of wet sieving and decanting (Daniels & Skipper, 1982), followed by density gradient centrifugation in a 50% sucrose solution (Brundrett, Bougher, Dell, Grove, & Malajczuk, 1996). The spores were pipetted onto a white gridded cellulose nitrate filter (1.2‐μm pore size), washed with distilled water, spread evenly, and counted under a compound microscope (Olympus BX51, Japan) at 10 × 10 magnification. The AM fungal spore density was expressed on a soil dry weight basis.

### 2.5 AM fungal colonization

Subsamples of frozen roots were cleaned for 30 min in 10% KOH at 90°C, bleached in alkaline hydrogen peroxide for 20 min, acidified in 1% HCl for 3 min, and stained with trypan blue in lactophenol (Phillips & Hayman, 1970). AM fungal colonization was measured using the gridline intersect method described by Giovannetti and Mosse (1980) under a compound microscope at 10 × 20 magnification. The presence of arbuscules, vesicles, or hyphae was recorded at each point where the roots intersected a line.

### 2.6 Shoot and root nutrient contents

Dry shoot and root materials were ground in a Wiley mill, passed through a 1.0‐mm or 0.5‐mm mesh screen, and stored at room temperature prior to the laboratory analyses. A 0.2‐g subsample was passed through a 0.5‐mm mesh screen and mineralized in concentrated sulfuric acid as described by Isaac and Johnson (1976) before its N, P, and K contents were quantified using the methods described by Jackson (2005). A 1.0‐g subsample was passed through a 1.0‐mm mesh screen and weighed for dry ashing at 525–550°C before the plant tissue Cu, Zn, Fe, Mn, Ca, and Mg contents were determined using FAAS (AA‐7003A, Beijing, China) according to Jorhem's (2000).

### 2.7 Statistical analyses

All data were subjected to a Bartlett's test and were transformed to achieve normality of distribution before analysis. Permutational multivariate analysis of variance (PERMANOVA) with 100 permutations and principal component analysis (PCA) were carried out using the function adonis() and rda() in the R package Vegan (Oksanen et al., 2013) to explore variations in soil properties, AM fungal attributes, and plant nutrient status by afforestation type. ANOVA was performed with the general linear model procedure (SAS Institute Inc. 2004) to test treatment effects on soil properties, AM fungal colonization rate, spore density, and nutrient contents of oriental arborvitae shoots. When treatment effects were significant (*p *<* *.05), treatment means were compared using Duncan's multiple range test, at the 5% level of significance. In order to select the variables which were significantly associated with tree performance, Pearson's correlation analysis was carried out to evaluate relationships between the afforestation‐type‐affected soil parameters, AM fungal attributes, plant nutrient status, and oriental arborvitae growth (SPSS 20.0, Chicago, IL, USA). After that, structural equation modeling (SEM) was used to show causal relationships between these variables (Taniguchi, Kanzaki, Tamai, Yamanaka, & Futai, 2007; Zheng et al., 2016), and the significance of the standardized regression weights was determined based on the Mantel test (Taniguchi et al., 2007; Zheng et al., 2016). SEM was performed using AMOS 17.0 (Amos Development Corporation, Meadville, PA, USA) (Zheng et al., 2016).

## 3 RESULTS

### 3.1 Soil properties

**Figure 1:**
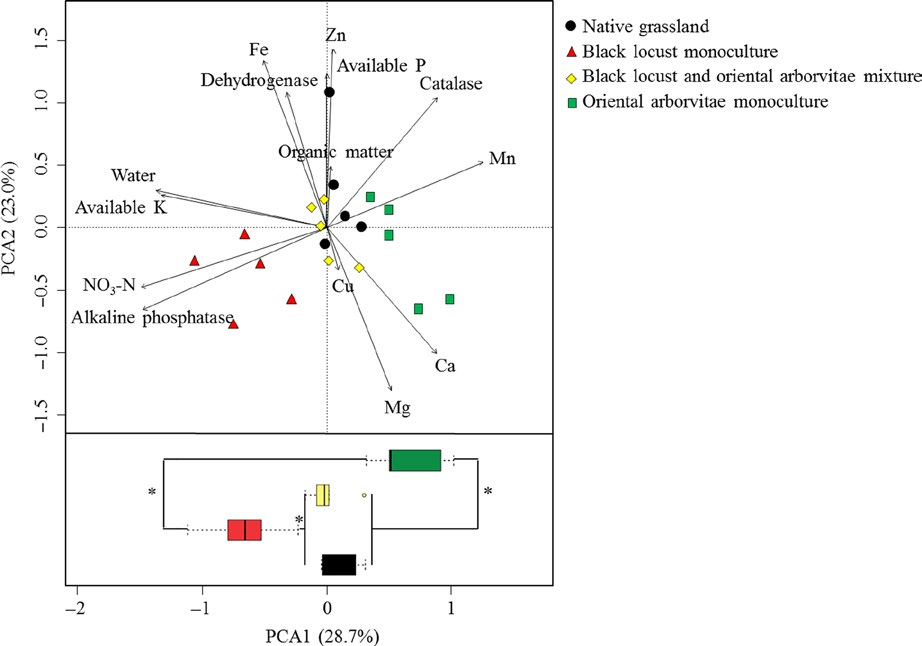
Ordination of afforestation types from a principal component analysis (PCA) of soil properties, including soil water, organic matter, NO_3_‐N, available P, available K, Cu, Zn, Fe, Mn, Ca, Mg, catalase, alkaline phosphatase, and dehydrogenase. Points represent the PCA1 and PCA2 scores. *: the difference is significant according to Duncan's test (*n *=* *5, *p *<* *.05). Box plots show the frequency and intensity of soil properties from different afforestation types. *Box length* represents interquartile range; *vertical line in box,* group median; and *whiskers,* group minimum and maximum values

Soil property data varied significantly (PERMANOVA, *Pseudo‐F *=* *3.39, *p *<* *.05) by afforestation type. The PCA also showed that soil property data were structured by afforestation type (Figure 1). Along Axis 1, which accounted for 28.7% of the variation, soil properties under the black locust × oriental arborvitae mixture were significantly different from soil properties under monocultures of these species but were similar to those of the native grassland. Among these selected soil parameters for PCA, contents of soil water, NO_3_‐N, available K and Mn, and activities of catalase and alkaline phosphatase were significantly affected by afforestation type (Table 1). Compared with the native grassland, black locust monoculture increased soil NO_3_‐N and available K contents and alkaline phosphatase activity and reduced soil Mn content and catalase activity, and oriental arborvitae monoculture reduced soil water and available K contents and catalase activity and increased soil Mn content. All the values of the afforestation‐type‐affected soil parameters in the black locust × oriental arborvitae mixture were between those of their monoculture.

**Table 1:** Effects of afforestation types on soil properties

Afforestation type	Water (%)	Organic matter (g/kg)	NO_3_‐N (mg/kg)	Available P (mg/kg)	Available K (mg/kg)	Cu (mg/kg)	Zn (mg/kg)	Fe (mg/kg)	Mn (mg/kg)	Ca (g/kg)	Mg (g/kg)	Catalase (mg g^−1^ min^−1^)	Alkaline phosphatase (mg g^−1^ h^−1^)	Dehydrogenase (mg ml^−1 ^h^−1^)
Native grassland	19.79 a	9.03 a	10.48 c	88.34 a	85.32 b	0.58 a	0.65 a	2.61 a	6.48 b	0.52 a	0.027 a	2.12 a	4.65 bc	0.20 a
Black locust monoculture	20.58 a	9.23 a	20.35 a	75.81 a	92.33 a	0.65 a	0.15 a	2.57 a	3.85 c	0.49 a	0.027 a	1.39 d	11.86 a	0.13 a
Black locust and oriental arborvitae mixture	18.25 b	10.98 a	12.22 b	85.96 a	91.69 a	0.65 a	0.25 a	2.37 a	7.90 ab	0.59 a	0.029 a	1.53 c	5.88 b	0.23 a
Oriental arborvitae monoculture	13.61 c	10.32 a	9.61 c	75.09 a	70.14 c	0.75 a	0.37 a	2.34 a	8.96 a	0.69 a	0.034 a	1.78 b	3.42 c	0.13 a
Effects of afforestation type (*P* value)	<0.001	ns^a^	<0.001	ns	0.049	ns	ns	ns	0.001	ns	ns	<0.001	<0.001	ns

Means with different letters for the same soil parameter are significantly different according to Duncan's test (*n *=* *5, *p *<* *.05).

a ns, not significant (*p *≥* *.05).

### 3.2 AM fungal colonization and spore density

**Figure 2:**
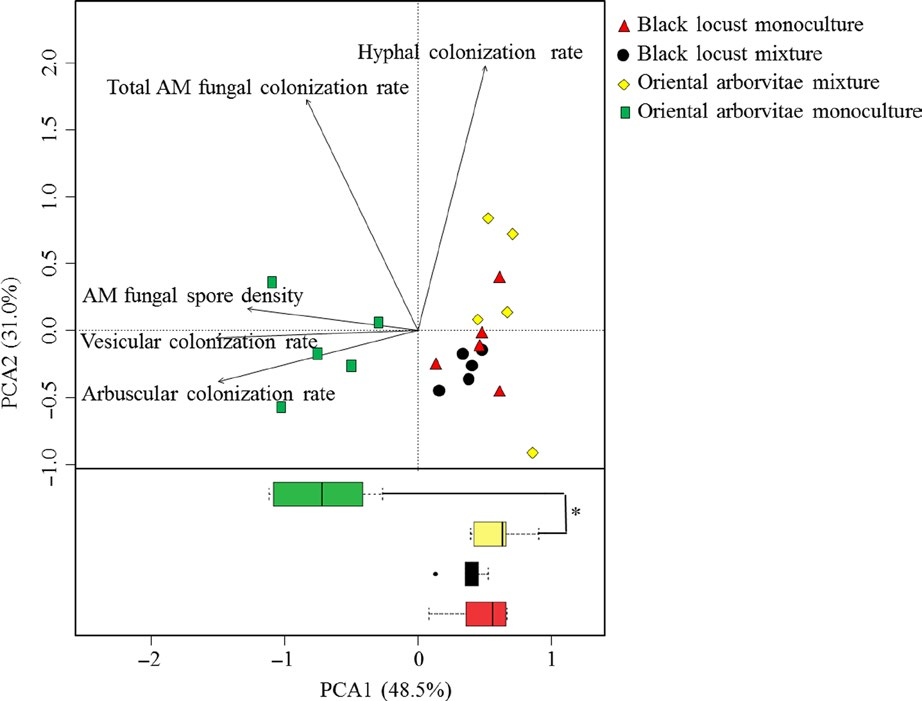
Ordination of afforestation types from a principal component analysis (PCA) based on arbuscular mycorrhizal (AM) fungal colonization and spore density. Points represent the PCA1 and PCA2 scores. *: the difference is significant according to Duncan's test (*n *=* *5, *p *<* *.05). Box plots show the frequency and intensity of AM fungal attributes from different afforestation types. *Box length* represents interquartile range; *vertical line in box,* group median; and *whiskers,* group minimum and maximum values

The AM fungal attributes varied significantly (PERMANOVA, *Pseudo‐F *=* *11.90, *p *< .01) by afforestation type. The PCA further revealed that the AM fungal attributes under oriental arborvitae in monoculture were significantly different from that in mixture with black locust, but the AM fungal attributes under black locust in monoculture were not different from that in mixture with oriental arborvitae (Figure 2). AM fungal spore density under black locust and all measurements of AM fungal colonization of black locust roots were similar regardless of whether oriental arborvitae was present. However, AM fungal spore density under oriental arborvitae and the colonization rate of oriental arborvitae roots by vesicles, arbuscules, and AM fungal structures as a whole were significantly higher in monoculture than in mixture with black locust (Table 2).

**Table 2:** Arbuscular mycorrhizal (AM) fungal spore density, colonization rate, and nutrient contents in the shoots of black locust and oriental arborvitae in monoculture and in mixture

	Black locust			Oriental arborvitae		
	Monoculture	Mixture	*p* value	Mixture	Monoculture	*p* value
AM fungal spore density (g^−1^ dry soil)	6.66 a	4.82 a	ns^a^	5.13 b	8.91 a	.001
AM fungal colonization rate (%)						
Total	66.62 a	70.08 a	ns	68.13 b	76.89 a	.048
Arbuscule	2.59 a	3.12 a	ns	1.56 b	4.82 a	.005
Vesicle	5.42 a	8.29 a	ns	10.39 b	25.80 a	<.001
Hyphae	46.01 a	39.31 a	ns	49.92 a	38.51 a	ns
Nutrient contents in shoots (g/kg)						
K	2.14 a	1.97 a	ns	4.56 a	4.78 a	ns
N	5.96 a	5.96 a	ns	5.07 a	5.17 a	ns
P	0.76 a	1.06 a	ns	1.22 a	1.41 a	ns
Cu	0.01 a	0.01 a	ns	0.01 a	0.003 a	ns
Zn	0.05 b	0.08 a	.014	0.05 a	0.05 a	ns
Fe	0.53 a	0.52 a	ns	0.47 a	0.41 a	ns
Mn	0.03 a	0.03 a	ns	0.06 b	0.09 a	.001
Ca	18.08 a	18.10 a	ns	31.66 b	44.42 a	.002
Mg	1.52 a	1.39 a	ns	1.26 b	1.90 a	.027

Means with different letters for the same parameter of black locust or oriental arborvitae are significantly different according to Duncan's test (*n *=* *5, *p *<* *.05).

a ns, not significant (*p *≥* *.05).

### 3.3 Plant nutrient status

**Figure 3:**
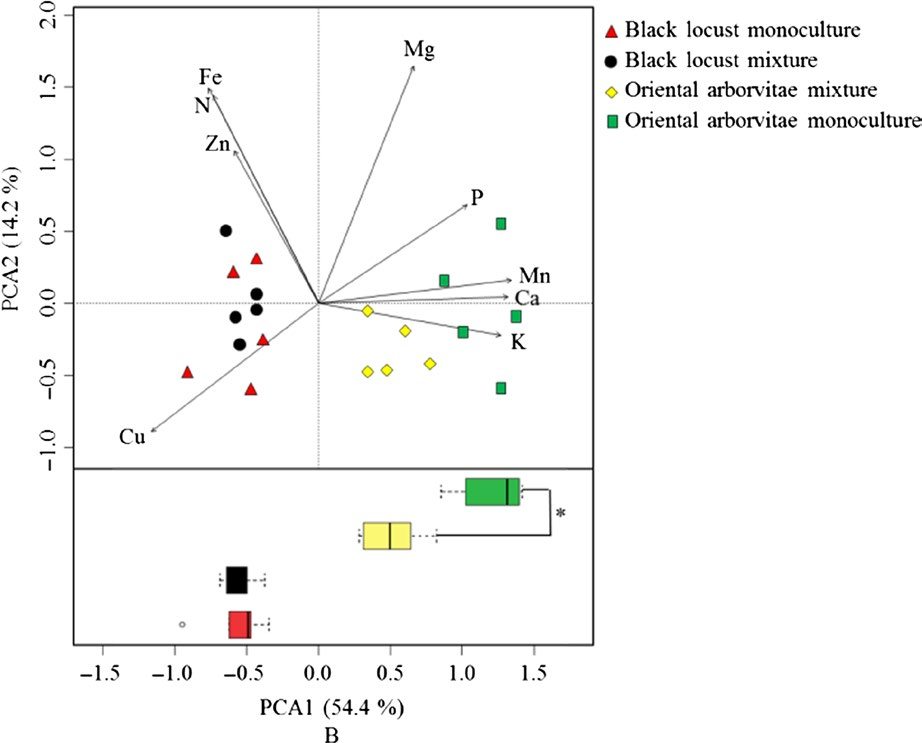
Ordination of afforestation types from a principal component analysis (PCA) based on nutrient status, that is, on N, P, K, Cu, Zn, Fe, Mn, Ca, and Mg contents in tree shoots. Points represent the PCA1 and PCA2 scores. *: the difference is significant according to Duncan's test (*n *=* *5, *p *<* *.05). Box plots show the frequency and intensity of nutrient status from different afforestation types. *Box length* represents interquartile range; *vertical line in box,* group median; and *whiskers,* group minimum and maximum values

The nutrient status of tree shoots varied significantly (PERMANOVA, *Pseudo‐F *=* *11.90, *p *< .01) by afforestation type, while that of tree roots did not (PERMANOVA, *Pseudo‐F *=* *1.74, *p *=* *.23). The PCA further showed that afforestation types could not be differentiated by PCA based on tree root nutrient status, but PCA based on the nutrient status of oriental arborvitae shoots revealed that afforestation type had an effect on oriental arborvitae (Figure 3). Among the nutrient parameters selected for PCA, the Mn, Ca and Mg contents of oriental arborvitae shoots differed by afforestation type, and the nutrient values of these shoots were higher in monoculture than in mixture (Table 2).

### 3.4 Plant growth

**Figure 4:**
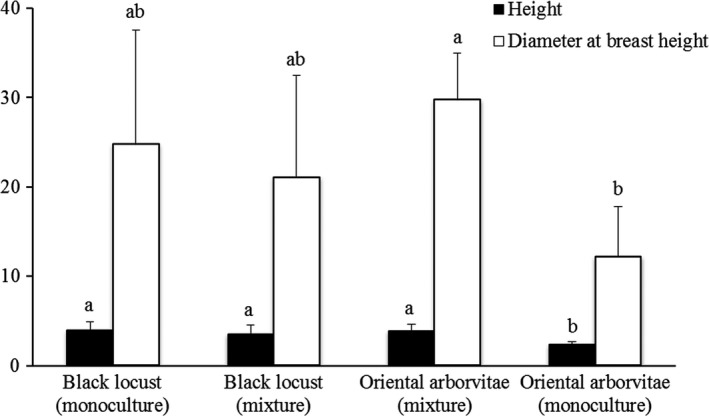
Height and diameter at breast height of black locust and oriental arborvitae growing in monoculture and in mixture. Means with different letters for the same parameter indicate significant differences according to Duncan's test (*n *=* *5, *p *<* *.05). The unit of height and diameter at breast height was m and cm, respectively

The presence of black locust increased the growth of oriental arborvitae, as shown by the increased mean height and diameter at breast height of oriental arborvitae in mixture compared with monoculture, while the growth of black locust was similar in mixture and in monoculture (Figure 4).

### 3.5 Relationships between the growth of oriental arborvitae, soil properties, AM fungal attributes, and plant nutrient status

**Figure 5:**
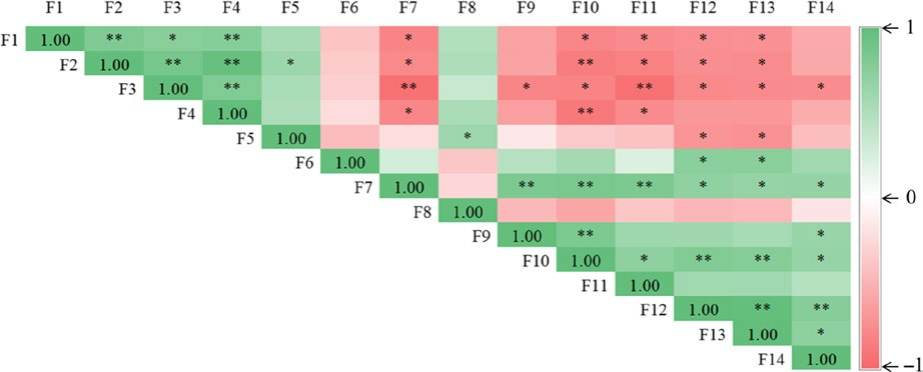
Pearson correlations between oriental arborvitae height, diameter at breast height, arbuscular mycorrhizal (AM) fungal attributes, soil properties and plant nutrient status. *: significant at *p *<* *.05; **: significant at *p *<* *.01. F1–14 represent oriental arborvitae height (F1), diameter at breast height (F2), soil water (F3), soil NO_3_‐N (F4), soil available K (F5), soil Mn (F6), catalase (F7), alkaline phosphatase (F8), arbuscular colonization rate (F9), vesicular colonization rate (F10), AM fungal spore density (F11), shoot Mn (F12), shoot Ca (F13), and shoot Mg (F14), respectively

Correlation analysis of the parameters affected by the afforestation type revealed that the growth of oriental arborvitae (height and diameter at breast height) was positively associated with soil water, NO_3_‐N, and available K contents, negatively correlated with soil catalase activity, vesicular colonization rate, AM fungal spore density, shoot Mn, and Ca contents, but unrelated with arbuscular colonization rate, alkaline phosphatase activity, and contents of soil Mn and shoot Mg (Figure 5).

**Figure 6:**
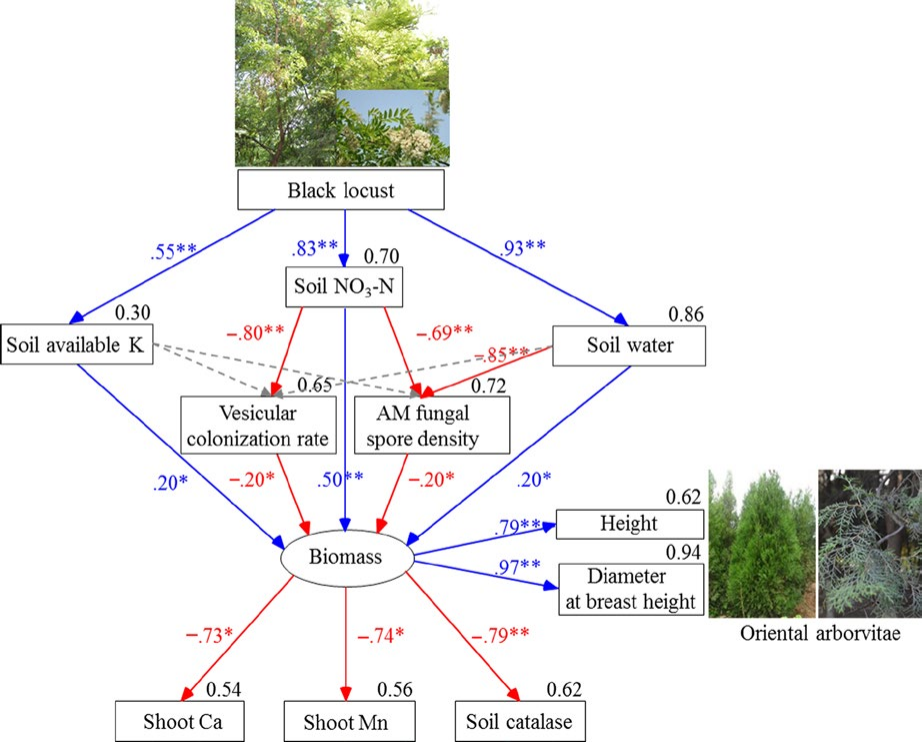
Structural equation model of relationships among oriental arborvitae height, diameter at breast height, vesicular colonization rate, AM fungal spore density, soil water, soil NO_3_‐N, soil available K, soil catalase, shoot Ca and Mn based on the result of Mantel statistics (*p *=* *.062, goodness‐of‐fit index = 0.93). The number on the top right corner of the soil, plant, or AM fungal parameters is the squared multiple correlations, and the number on the lines among these parameters is the standardized regression weights (*: significant at *p *<* *.05; **: significant at *p *<* *.01). Red solid lines: the standardized regression weights are negative; blue solid lines: the standardized regression weights are positive; gray dashed lines: the standardized regression weights are not significant (*p *≥* *.05), and thus, their statistics are not shown

Structural equation models indicated that (1) black locust in mixture improved soil NO_3_‐N, available K, and water contents, and this improvement directly increased oriental arborvitae growth; (2) the increase in soil NO_3_‐N reduced AM fungal spore density and the vesicular colonization rate, and the increase in soil water reduced AM fungal spore density; (3) the decrease in AM fungal spore density and the vesicular colonization rate indirectly enhanced oriental arborvitae growth because the production of spores and vesicles adversely affects oriental arborvitae growth; and (4) the enhanced growth of oriental arborvitae in mixture further diluted shoot Ca and Mn and reduced soil catalase activity (Figure 6). All of these results suggest that mixing black locust with oriental arborvitae improved soil water and fertility (NO_3_‐N and available K), that this improvement directly or indirectly enhanced oriental arborvitae growth, and that this enhanced growth in turn reduced soil catalase activity and shoot Ca and Mn contents.

## 4 DISCUSSION

Our results showed that oriental arborvitae grows better in the presence of black locust, which is in agreement with the findings of Forrester et al. (2005). As hypothesized, the benefit of mixed planting can stem from improvements in soil properties. In this study, we found that the soil properties under the black locust × oriental arborvitae mixture differed from those under monocultures of these trees, whereas the soil properties under the mixed stand were similar to those of the native grassland. This means that monocultures of black locust and oriental arborvitae cause a change in soil properties, whereas mixed stands tend to maintain soil ecological stability, as supported by the findings of other authors (Khanna, 1997; Liu et al., 2009). More specifically, black locust mixed with oriental arborvitae improved soil water, NO_3_‐N and available K levels, and this improvement significantly enhanced oriental arborvitae growth based on the results of correlation and SEM analysis.

Surface soil water content under the mixed stand was higher than under oriental arborvitae monocultures but lower than under black locust monocultures, suggesting the existence of complementary functions between black locust and oriental arborvitae resulting from hydraulic lifting. As we know, the root system of oriental arborvitae is fibrous and shallow, while black locust has a taproot and long oblique lateral roots penetrating deep in the soil (Zhai, Yin, & Zhang, 1991). Thus, the deep root system of black locust can lift water stored in deep soil layers and redistribute it closer to the surface (Caldwell, Dawson, & Richards, 1998). On the other hand, due to big canopy and low transmittance in mixture, the transpiration of oriental arborvitae reduced in the presence of black locust (Zhai et al., 1991). All these reasons maybe explain why soil water content under the black locust × oriental arborvitae mixture was higher than in oriental arborvitae monocultures. This higher availability of water in mixture could stimulate oriental arborvitae growth.

It is generally accepted that N‐fixing species can improve soil N pools and thus benefit adjacent non‐nitrogen‐fixing species (Boring & Swank, 1984; Olesniewicz & Thomas, 1999; Rice et al., 2004; Rothe & Binkley, 2001; Tateno et al., 2007). In this study, we too found that the soil NO_3_‐N content under the black locust × oriental arborvitae mixture was higher than under the oriental arborvitae monoculture. Soil N accrual can increase soil available K content through the displacement of K^+^ by ${\text{NH}}_{4}^{+}$ ions between the platelets of silicate clays (Duan, Huang, & Zhang, 2016; Kang et al., 2001; Wang, Liu, & Xue, 2012; Zhang et al., 2003). Such mobilization of K by a raising N level may explain why we found the same influence of afforestation type on soil N and K, in this study. The increase in soil available K under black locust monocultures could explain why soil available K under the mixed stand was higher than under the oriental arborvitae monoculture, even though oriental arborvitae monocultures reduce soil available K levels. Consequently, black locust mixed with oriental arborvitae can increase soil N and K fertility and this higher soil fertility greatly benefits the growth of oriental arborvitae.

Soil enzymes are critical for ecosystem functioning, and soil enzyme activity can indicate changes in soil metabolic functions and nutrient cycling (Saha, Prakash, Kundu, Kumar, & Mina, 2008). Soil alkaline phosphatase is an inducible enzyme involved in the mobilization of organic P, an activity that was affected by afforestation type in our study. The highest activity of soil alkaline phosphatase was under black locust monoculture, the lowest was under oriental arborvitae monoculture, and the value in the black locust × oriental arborvitae mixture was between that of their monoculture, indicating that P demand differed with tree species and thus mixtures of black locust and oriental arborvitae may improve soil P utilization efficiency. Catalase breaks down H_2_O_2_, protecting plants from oxidative stress (Rhee, Yang, Kang, Woo, & Chang, 2005). We found greater soil catalase activity in oriental arborvitae monoculture than in black locust monoculture and found soil catalase activity levels in mixed black locust and oriental arborvitae stands lying between the levels found in their monocultures, which is consistent with the results of Li, Li‐gang, and Hong‐yue (2003). Our SEM results indicate that soil catalase activity was dependent on tree growth, with active growth of oriental arborvitae co‐occurring with low levels of soil catalase activity, which may suggest that black locust mixed with oriental arborvitae reduces stress levels in the soil system and thus benefits the growth of oriental arborvitae.

It has been found that N‐fixing species are able to not only enhance soil fertility but also affect the plant nutrition status of adjacent non‐nitrogen‐fixing species (Hu et al., 2017). For instance, Persian walnut is considered as a non‐nitrogen‐fixing species that exhibits weak N uptake capability grown in monoculture (Chifflot, Bertoni, Cabanettes, & Gavaland, 2006; Loewenstein & Pallardy, 2002), whereas its N nutrition can be greatly improved if interplanted with suitable N‐fixing nurse tree species such as black locust (Bohanek & Groninger, 2005; Clark, Hemery, & Savill, 2008; Jose, Williams, & Zamora, 2006). In this study, we found black locust mixed with oriental arborvitae had a significant influence on the nutrient status of oriental arborvitae shoots, but not on their root nutrient status. Specifically, black locust mixed with oriental arborvitae significantly reduced Mn, Ca, and Mg contents of oriental arborvitae shoots compared to oriental arborvitae monoculture, and the primary differences in Mn and Ca contents were attributed to the enhanced growth of oriental arborvitae in mixture based on our correlation and SEM results.

It is well known that AM symbiosis varies with environmental conditions (An et al., 2010; Entry, Rygiewicz, Watrud, & Donnelly, 2002; Grant, Bittman, Montreal, Plenchette, & Morel, 2005; Herrera, Salamanca, & Barea, 1993). Here, the lower total AM fungal root colonization and spore density in the rhizosphere of oriental arborvitae growing in mixture may reflect black locust in mixture suppress the development of AM symbiosis in oriental arborvitae roots. AM fungi provide water and nutrients to the host plant mitigating stress, but the symbiosis has a high energetic cost (Smith & Read, 2008; Taniguchi et al., 2007). To prevent AM fungi from becoming a burden on well‐fed plants (Smith & Read, 2008), high soil fertility triggers the repression of AM symbiotic development in roots (Pagano, Scotti, & Cabello, 2009). As discussed above, black locust mixed with oriental arborvitae improved soil fertility (NO_3_‐N and K) and water conservation. Our SEM results further indicated that these higher soil water and NO_3_‐N contents in mixture induced a decrease in vesicular colonization rate and AM fungal spore density in the rhizosphere of oriental arborvitae. It has been found that the AM fungal spores and vesicles are storage or resting structures involved in “long‐term” survival and producing them probably comes with a high carbon cost (Duckmanton & Widden, 1994; Kubota, McGonigle, & Hyakumachi, 2005; Torrecillas, del Mar Alguacil, & Roldán, 2012). Thus, the lower colonization rate of oriental arborvitae roots by vesicles and AM fungal spore density in the rhizosphere of oriental arborvitae in response to increased soil N fertility and water conservation in the mixed stand benefit the growth of oriental arborvitae.

## 5 CONCLUSIONS

This study found that mixing oriental arborvitae with black locust modifies soil properties, the development of AM symbiosis, and plant nutrition status, and these modifications could explain why oriental arborvitae grows better in mixture than in monoculture. Specifically, the following mechanisms appear to support enhanced oriental arborvitae growth: (1) an increase in soil water, and N and K availability in the presence of black locust, directly stimulating the growth of nearby oriental arborvitae trees; (2) a reduced production of AM fungal vesicles and spores through black locust‐related raise in N fertility and water conservation, thus freeing up carbon to fuel plant growth; and (3) nonlimiting soil enzyme activity and shoot nutrient status in oriental arborvitae. In conclusion, a black locust × oriental arborvitae mixture planted in the Loess Plateau can help meet a wide range of economic, silvicultural, and sustainability objectives. However, this finding is only based on 10‐year‐old plantation. Maybe, this mixture efficacy at supporting plant growth will change as trees age, and thus, it is necessary to conduct further studies on a long‐term interaction in this mode of mixture.

## CONFLICT OF INTEREST

None declared.

## AUTHOR CONTRIBUTIONS

Min Sheng conceived and designed the study. Xuedong Chen, Xinlu Zhang, and Wei Li performed the experiments. Min Sheng and Ming Tang performed the data analyses. Ming Tang and Xuedong Chen wrote the article. Chantal Hamel and Ming Tang reviewed and edited the manuscript. All authors read and approved the manuscript.
